# Xin-Ji-Er-Kang protects heart from ischemia-reperfusion injury by rebalancing lipid metabolism

**DOI:** 10.3389/fphar.2022.981766

**Published:** 2022-08-23

**Authors:** Li-Jun Sun, Xiao-Yu Wang, Jie Xia, Yan-Mei Xu, Yu-Feng Liao, Yuan-Yuan Qin, Xue-Wan Ge, Pei-Wen Zhao, Tong Xu, Xiao-Ling Zhu, Shan Gao, Rui Xiao, Xue-Sheng Liu, Kai Zhou

**Affiliations:** ^1^ Department of Pharmacology, School of Basic Medical Sciences, Anhui Medical University, Hefei, China; ^2^ Department of Anesthesiology, The First Affiliated Hospital of Anhui Medical University, Hefei, China; ^3^ Ipswich Hospital, East Suffolk and North Essex NHS Foundation Trust, Ipswich, United Kingdom

**Keywords:** anti-inflammatory effect, lipid metabolism, lipidomics, Xin-Ji-Er-Kang, myocardial ischemia-reperfusion injury

## Abstract

**Background and Purpose:** We have previously reported a cardioprotective effect with Xin-Ji-Er-Kang (XJEK) treatment in mice with myocardial infarction (MI)-induced heart failure, but no report about its potential functions in myocardial ischemia-reperfusion (MIR) injury. Here we studied the therapeutic effects of XJEK on MIR injury and investigated the mechanisms involved.

**Experimental Approach:** MIR model of Balb/c mice induced by left anterior descending coronary artery ligation for half an hour, followed by reperfusion, was utilized to study the potential therapeutic effects of XJEK on MIR-induced cardiac injury. Ultra-performance liquid chromatography tandem Orbitrap mass spectrometry platform was used for studying serum lipid metabolic signatures.

**Key Results:** MIR caused cardiac dysfunctions, cardiac injury, myocardial fibrosis, and increased inflammation, and all the observed abnormalities caused by MIR were largely corrected by XJEK treatment. Mechanistically, XJEK exerts its cardioprotective effect in the context of MIR injury by suppressing MIR-induced inflammation and dysregulation of serum lipid metabolism.

**Conclusion and Implications:** We have demonstrated for the first time that XJEK protects heart from MIR injury by restoring dysregulated lipidomics. Our data provide new evidence to support a therapeutic effect for XIEK on MIR-induced cardiac injury, and pave the way for exploring the therapeutic potential of XJEK in large animal study and early clinical trial.

## 1 Introduction

Despite that the annual death rate attribute to coronary heart diseases (CHD) had declined 28.1% from 2007 to 2017 due to the increasing successful use of interventional coronary reperfusion strategies including percutaneous coronary intervention (PCI), cardiovascular diseases remain the leading cause of death worldwide (Virani et al., 2020). Although early thrombolytic therapy and primary PCI are the most effective strategies for restoring blood flow to the ischemic myocardium and improving the clinical outcome after acute myocardial infarct (MI), such interventional processes also cause cardiac injury, namely myocardial ischemia-reperfusion (MIR) injury (Hausenloy and Yellon, 2013). Subsequently, extensive studies aiming at uncovering the underlying molecular mechanism of MIR injury have been conducted in order to identify an effective therapy for preventing MIR injury (Xu et al., 2019). However, there still a big challenge to translate the preclinical findings of infarct size reduction into better clinical outcomes on patients.

Both the inflammatory reactions and dysregulated cell metabolism have been suggested as the key underpinning pathological causes of MIR injury (Goetzman et al., 2022; Li et al., 2022). It has been reported that alteration of fatty acid metabolism occurs in cardiomyocytes under hypoxia and reoxygenation condition (Ford, 2002). Lipids serves not only as a store of fatty acids for energy production, but also as the cellular building blocks for the synthesis of other signaling intermediates molecules. Therefore, it is plausible to think that lipid metabolic disorder may play a key role in MIR injury. Indeed, elevated circulating triacylglycerols (TG) and very-low-density lipoprotein (VLDL) were observed in patients who suffered MIR injury (Kjellqvist et al., 2016). However, a comprehensive investigation into the lipid metabolism after MIR injury has not been reported yet.

Xin-Ji-Er-Kang (XJEK) is a traditional Chinese formula with a clinically proven cardiovascular protective effect for CHD patients. In previous studies, we have also reported a cardioprotective effect with XJEK treatment in mice with MI-induced heart failure (Hu et al., 2018; Cheng et al., 2019). The protective effect of XJEK was considered to be connected with the endothelial dysfunction and oxidative stress. However, little is known about a potential therapeutic effect of XJEK on MIR injury. Moreover, a comprehensive lipidomic profile after MIR injury remains to be determined. In this study, MIR mice model induced by the left anterior descending coronary artery ligation and reperfusion was used to investigate the potential therapeutic effects of XJEK on MIR. Serum lipidomics, which has been recently developed platform with a power to determine the global changes of different lipids in eukaryotes including sphingolipids, glycerophospholipids, glycerolipids and non-esterified fat (Züllig et al., 2020), was performed to uncover the global changes of lipid contents and metabolic pathways after MIR injury with or without XJEK treatment. We found that XJEK could protect heart from MIR injury, and partially restore the dysregulated lipid metabolism induced by MIR injury.

## 2 Materials and methods

### 2.1 Animal experiments

A total of 50 male Balb/c mice weighing 28–30 g, aged 8 weeks, were purchased from Shanghai Slac Laboratory Animal Corp. Ltd. [Certificate No. SCXK (JING) 2016–0260]. A MIR model was established by following steps: the mice were anesthetized with pentobarbital sodium at the dose of 45 mg/kg, and ventilated with positive pressure via a tube inserted into the trachea which connected to a small animal respirator (BL420S, Chengdu Techman Software Co.,Ltd., China). After the animal chest was shaved, a left thoracotomy was conducted in the intercostal space of the apex beat, and then the heart was fast exposed. The left anterior descending coronary artery was ligated with a 7–0 thread for 30 min. Subsequently, the ligation line was untied to recover blood supply.

After MIR surgery, survived mice were randomly divided into 4 groups: sham, MIR, MIR/XJEK, and MIR/fosinopril groups. For sham/control group, mice underwent the same surgical procedure, but without ligation of left coronary artery, and received an intragastric gavage of distilled water. For MIR, MIR/XJEK, and MIR/fosinopril (positive control) groups: after MIR surgery, mice were randomly received an intragastric gavage of distilled water, XJEK (8 g/kg/day), and fosinopril (2 mg/kg/day), respectively, for 6 weeks. XJEK decoction was prepared by decocting original materials, then spray dried for long-term store, and suspended with water before gastric gavage to animals. All animals were allowed free access to food or water, and fed under the controlled temperature (25°C and 12 h light-dark cycle). Body weight and food intake were checked weekly. All experimental steps were approved by the Committee on the Ethics of Animal Experiments of Anhui Medical University (No. 14–862).

### 2.2 Echocardiographic measurements

Mice were anesthetized intraperitoneally with 1% pentobarbital sodium and fixed on a tin foil board with medical tape. To monitor the change of electrocardiogram during the MIR surgery, mice were monitored with a BL-420S Data Acquisition & Analysis System (Chengdu TME Technology Co., Ltd., China) and electrocardiogram results were recorded 3 min preoperatively, 30 min postoperatively and 5 min post-reperfusion. Non-invasive tests of cardiac function were performed before the mice sacrificed. Cardiac function indicators such as left ventricular ejection fraction (LVEF), fractional shortening (LVFS), and internal diameter end diastole (LVIDd) and end systole (LVIDs) were collected using a Doppler ultrasound diagnostic instrument (VINNO 6vet) as previously reported (Yang et al., 2022).

### 2.3 Pathological examination

A portion of the heart (20%) was immersion-fixed in neutral 10% buffered formalin for histological analysis. Serial sections (5 μm) were subjected to hematoxylin and eosin (H&E) and masson staining analysis according to the standard protocol, followed by measurements of the myocyte cross-sectional area (CSA), collagen volume fraction (CVF) and perivascular collagen area (PVCA) using Image J.

### 2.4 Enzyme-linked immunosorbent assay

Serum NT-proBNP, cTnI, IL-1β and TNF-α levels were assessed using respective ELISA kits according to the manufacturers’ instructions, generally comply with the following procedures: Samples were diluted with dilution buffer, and added with HRP labeled antibody. All samples were incubated at 37°C before the reaction stopped. The OD valves of all samples were measured with Microplate Reader at 450 nm.

### 2.5 Serum lipidomics analysis

#### 2.5.1 Sample collection and preparation

Mouse blood was collected via orbital venous plexus before sacrificed, and stored in a refrigerator at −80°C until use. For serum lipidomic studies, 200 μl of serum was mixed with 200 μl of extractant (CH_3_OH:CHC_l4_ = 1:2, pre-chilled for 30 min at −20°C) with shaking at 1,200 rpm/min for 30 min at room temperature (RT). The mixture was incubated with 125 μl of ultrapure water on a shaker at 1,200 rpm/min at RT for additional 10 min. After span at 12,000 rpm/min at 4°C for 10 min, the lower organic phase of the aqueous and protein phases was transferred into a new EP tube, then blow dried with nitrogen. After then, 100 μl C_3_H_8_O and 100 μl C_2_H_3_N were pre-mixed and added to the EP tube to re-suspend the dried pellet. The sample was vortexed for 5 min and sonicated for another 5 min, then was filtered through a 0.22 μm filter membrane. Ten μl of the filtered solution from 9 samples were pooled together and used as quality control (QC) sample to monitor the system suitability, stability and mass accuracy in positive and negative ionization modes with ESI source.

#### 2.5.2 Chromatography and mass spectrometry analysis

Ultra-high performance liquid chromatography (UPLC) coupled with high resolution mass spectrometry (Q Exactive plus, Thermo Fisher, United States) was used to determine serum lipidomic profiles, with acetonitrile/water (60:40, containing 0.1% formic acid and 0.1% ammonium formate, v-v) used as mobile phase A, isopropanol/acetonitrile (90:10, 0.1% formic acid, 0.1% ammonium formate, v-v) as mobile phase B, and Acclaim C30 (3.0 μm, 2.1 mm*150 mm) as the Separation column, respectively. QC, along with other samples were repeatedly analyzed throughout the whole analytical sequence, with a scanning range from 100 m/z to 1,500 m/z and a resolution of 70,000 FWHW.

#### 2.5.3 Data processing and description

Xcalibur 4.1 software was used for peak identification, peak extraction, and lipid identification of lipid molecules and internal standard lipid molecules. For statistical description of metabolomics, standardized data were imported into SIMCA 14.1 software (Umetrics AB, Umeå, Sweden) for multivariate data analysis. Principal component analysis (PCA) was analyzed to provide a comprehensive view of clustering trends and outliers. The orthogonal projection to latent structure discriminant analysis (OPLS-DA) model was developed to further improve the resolution and validity of the model by filtering out the noise unrelated to the classification information. The variable importance for the projection (VIP) in the OPLS-DA model was used to determine biologically significant differential lipid molecules. In this experiment, VIP >1 and *p* < 0.05 of OPLS-DA were used as the screening criteria for significant differential lipid molecules. A 200-iteration permutation test was used to verify the robustness of the supervised OPLS-DA model and to assess the degree of overfitting.

### 2.6 Statistical analysis

The data are expressed as mean ± SD. For all the statistical analysis, multiple comparisons were performed by one-way ANOVA with the Tukey-Kramer exact probability test. Also, the least-squares method was applied for linear correlation between the selected variables. The *p* value of less than 0.05 was considered statistically significant.

## 3 Results

### 3.1 Xin-Ji-Er-Kang improves cardiac function after myocardial ischemia-reperfusion injury

**FIGURE 1:**
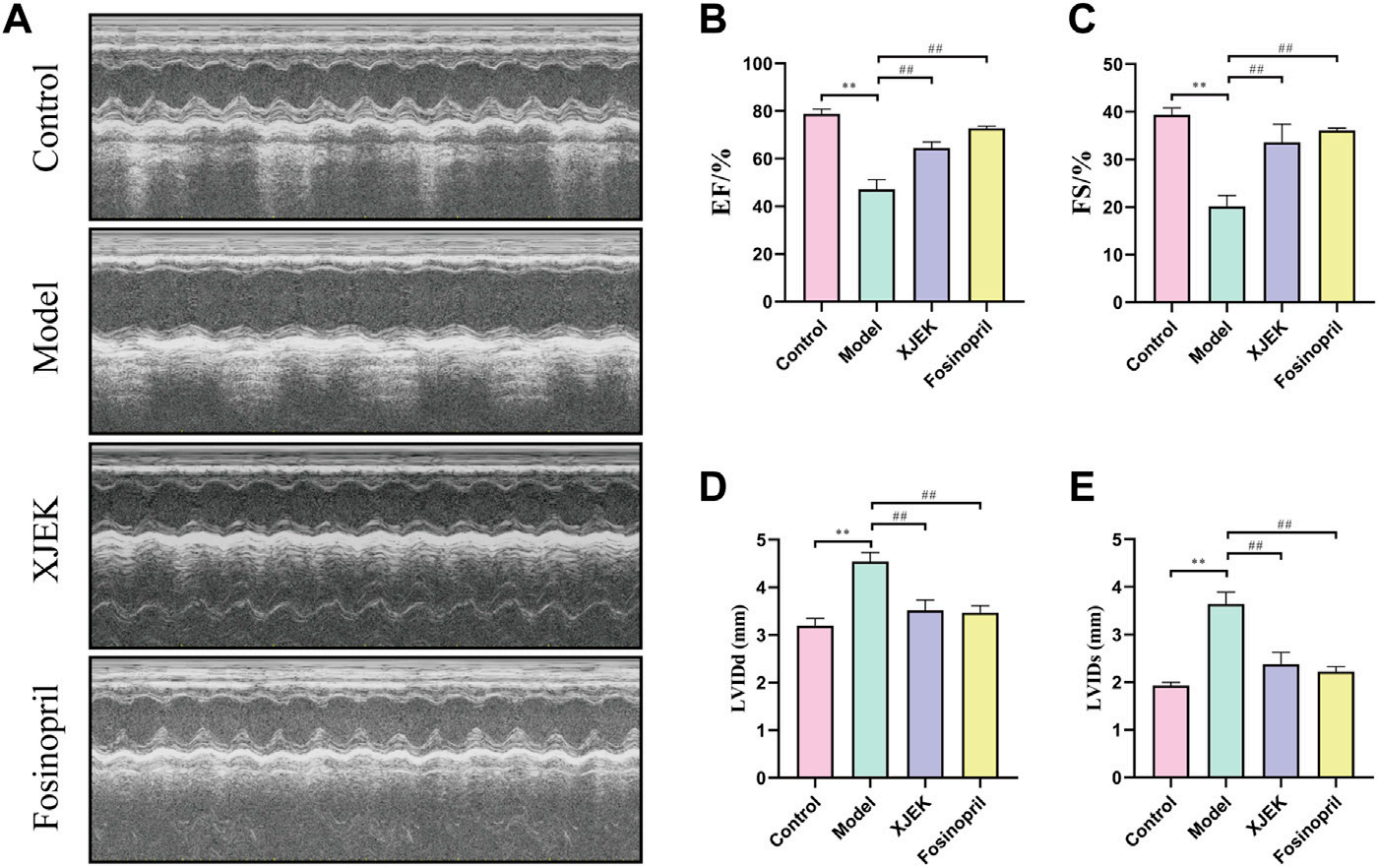
MIR-induced cardiac dysfunctions were reverted by XJEK. **(A)** Representative images of echocardiography with M-mode. Quantitative data of LVEF **(B)**, LVFS **(C)**, LVIDd **(D)** and LVIDs **(E)** from mice were presented here. ∗∗p *< 0.01* versus control group; ^*##*^ *p < 0.01* versus MIR model group. Data were expressed as mean ± SD (*n* = 6 mice for each group).

**FIGURE 2:**
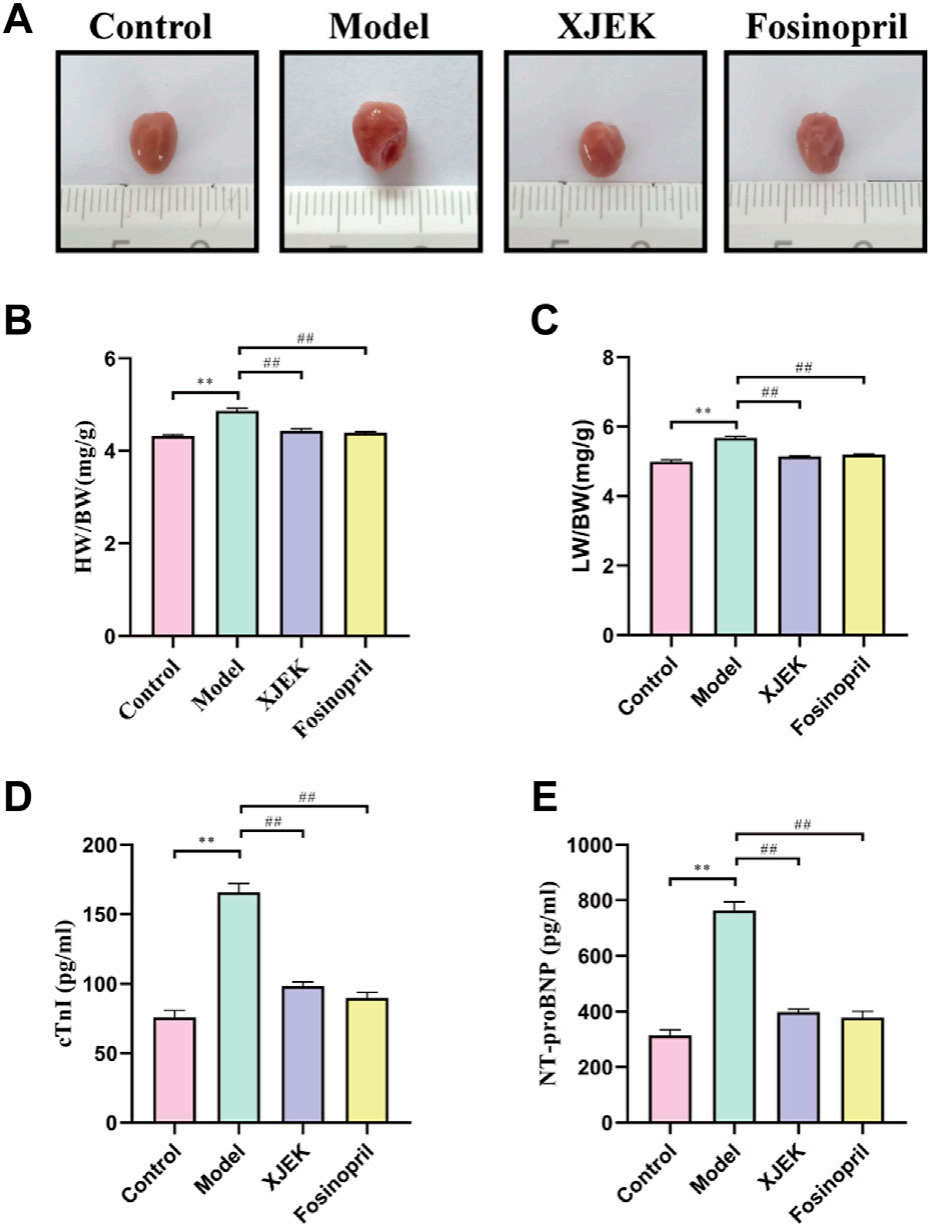
XJEK attenuated MIR-induced cardiac injury. **(A)** Pictures of the hearts from mice. **(B)** Ratios of heart weight to body weight (mg/g). **(C)** Ratios of lung weight to body weight (mg/g). Serum cTnI **(D)** and NT-proBNP **(E)** levels at 6 weeks post MIR. ^*∗∗*^ *p < 0.01* versus control group, ^*##*^ *p < 0.01* versus MIR model group. Data were expressed as mean ± SD (*n* = 6 mice for each group).

Echocardiography data showed that compared to sham control group the left ventricular diastolic and systolic diameters (LVIDd and LVIDs) were significantly increased, and left ventricular ejection fraction (EF) and left ventricular fraction shorting (FS) were significantly decreased in the MIR group (Figure 1), confirming MIR injury. Interestingly, the above-described MIR-induced cardiac dysfunctions were largely improved at the end of 6-week treatments with XJEK (Figure 1), showing an excellent therapeutic effect of XJEK, which is comparable with the positive treatment group (fosinopril). Moreover, increased ratios of heart/body weight (HW/BW, Figures 2A,B) and lung/body weight (LW/BW, Figure 2C) were observed in MIR groups compared with control group, with such increases were completely blunted by XJEK and fosinopril, respectively. Finally, ELISA results showed that the myocardial injury markers, serum NT-proBNP and cTnI, were significantly increased in the MIR group, with such elevations were reversed by both XJEK and fosinopril (Figures 2D,E). These data have collectively demonstrated that XJEK has a very good therapeutic effect on MIR injury.

### 3.2 XJEK reverses myocardial ischemia-reperfusion-induced adverse cardiac remodeling

**FIGURE 3:**
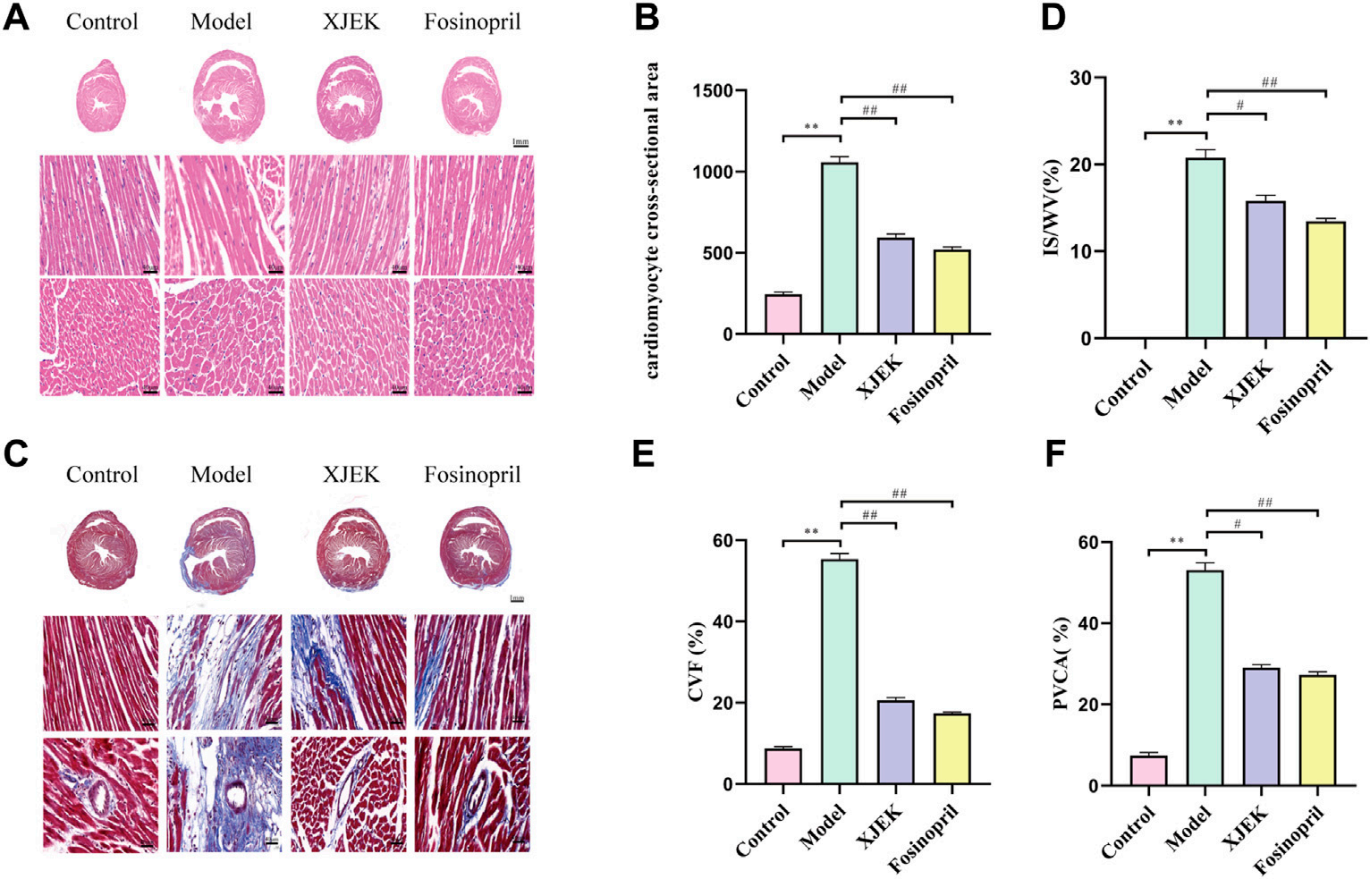
XJEK reduced MIR-induced cardiomyocyte hypertrophy and collagen deposition. **(A)** Representative images of myocardial slices with H&E staining. **(B)** Quantitative analyses of cardiomyocyte cross-section area. **(C)** Representative images of cardiac Masson-trichrome staining, with blue staining indicates infract area. Quantitative analyses of the scar size **(D)**, CVF **(E)** and PVCA **(F)** were presented. ∗∗*p < 0.01* versus control group, ^*##*^ *p < 0.01* versus MIR model group. Data were expressed as mean ± SD (*n* = 6 mice for each group).

H&E staining showed that compared with the control group MIR group displayed increased cross-sectional area (CSA) and longitudinal diameter of cardiomyocytes, indicative of cardiomyocyte hypertrophy, which was significantly attenuated by both XJEK and fosinopril (Figures 3A,B). Additionally, Masson-trichrome staining of the myocardium showed that MIR-induced infarct area was dramatically reduced by XJEK and fosinopril (Figures 3C,D). Finally, MIR-induced cardiac fibrosis was significantly attenuated by both drug interventions, as evidenced by that MIR-induced both myocardial collagen volume fraction (CVF) and perivascular collagen area (PVCA) were dramatically reduced by XJEK and fosinopril (Figures 3E,F). These data have collectively shown that XJEK attenuates MIR-induced adverse cardiac remodeling.

### 3.3 Xin-Ji-Er-Kang blunts myocardial ischemia-reperfusion induced inflammation

**FIGURE 4:**
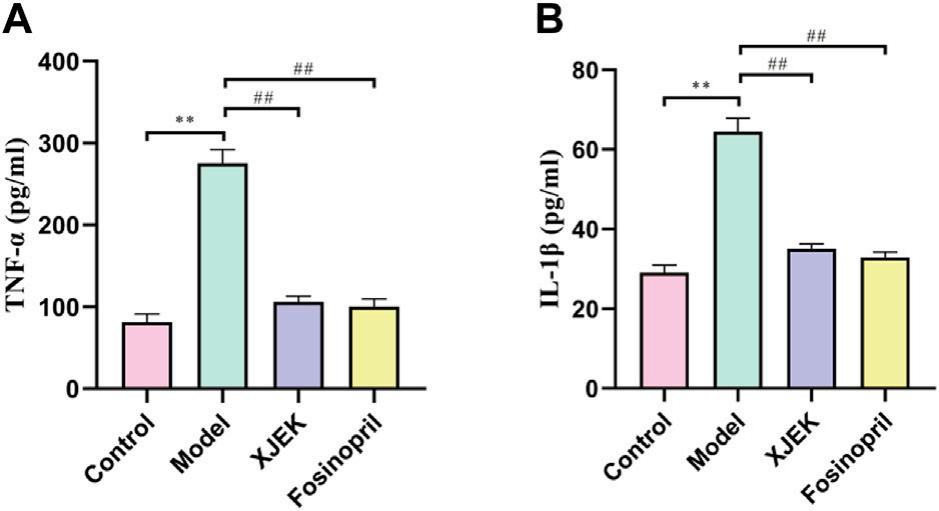
Serum levels of inflammatory cytokines. Serum TNF- α **(A)** and IL-1β **(B)** levels at 6 weeks post-MIR were presented here. *∗∗p* < 0.01 versus control group, ^*##*^ *p* < 0.01 versus MIR model group. Data were expressed as mean ± SD (*n* = 6 mice for each group).

As discussed early, inflammatory response is one of the key underlying pathological causes of MIR-induced cardiac dysfunctions and adverse remodeling. Moreover, we have so far demonstrated that XJEK could restore cardiac abnormalities induced by MIR injury. We therefore wondered if XJEK exerts such beneficial effects through modulating inflammation. Indeed, we observed that at 6 weeks after MIR surgery, both serum levels of TNF-α and IL-1β were significantly increased in MIR alone group (model group), while such increases were almost abrogated by both XJEK and fosinopril (Figure 4). Our data, therefore, suggest that XJEK restores MIR-induced cardiac dysfunctions and abnormalities at least partially through attenuating MIR-induced inflammation.

### 3.4 Xin-Ji-Er-Kang restores myocardial ischemia-reperfusion-induced dysregulation of serum lipidomic profiles

**FIGURE 5:**
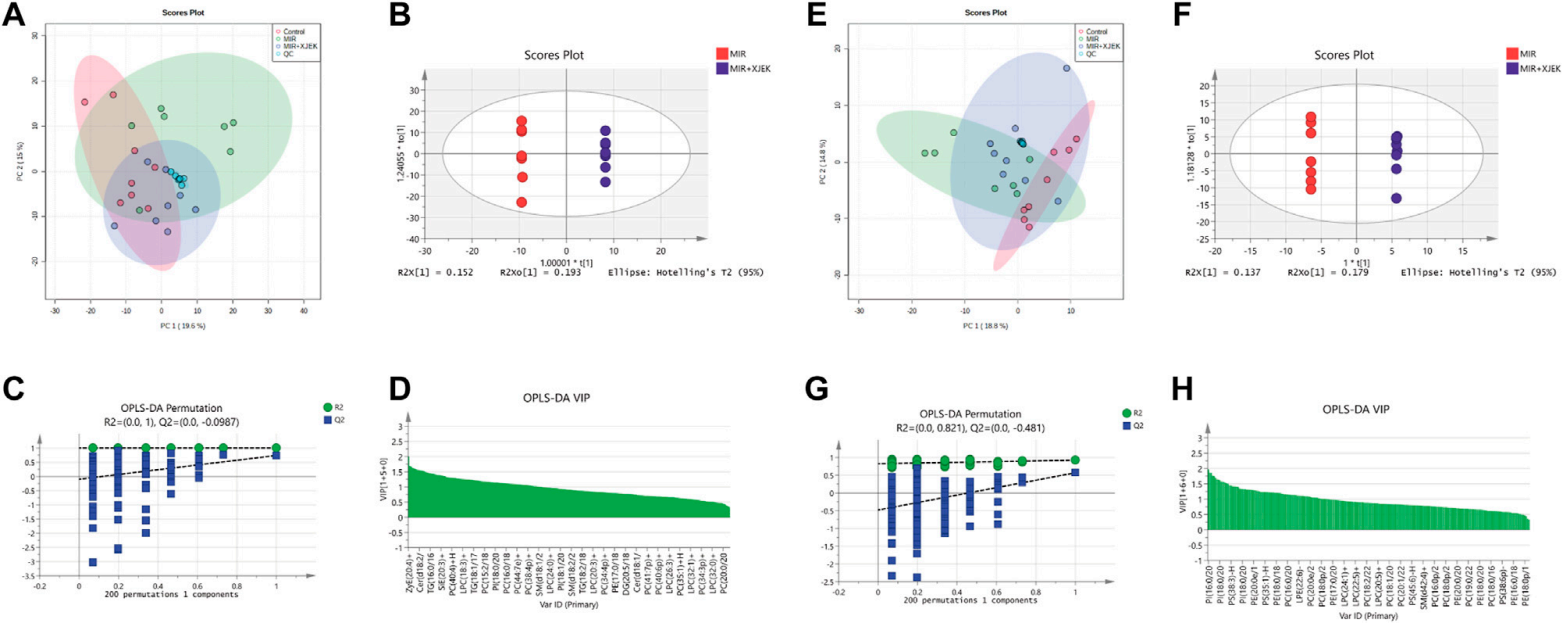
Serum lipid metabolic profiles. Serum lipid metabolic profiles were analyzed in both positive **(A–D)** and negative **(E–H)** model, respectively. **(A–E)** PCA analysis of lipid contents. **(B–F)** OPLS-DA analysis between MIR group and XJEK group. **(C–G)** Permutation test of OPLS-DA model. R2 represents the goodness of fit, and Q2 indicates the predictability of the models. **(D–H)** A variable importance of projection (VIP) plot with jack-knifed confidence intervals.

To further explore the possible mechanisms underlying the therapeutic effects of XJEK on MIR injury, serum lipidomic profiles in mice received shame surgery (control), MIR surgery alone, and MIR surgery plus XJEK treatments, respectively, were analyzed using UPLC-MS based lipidomic platform. We first checked sample grouping trends and outliers by conducting an unsupervised PCA analysis of serum lipid contents in positive mode. The PCA scoring plots in positive model showed a tight aggregation of quality control (QC) samples, showing a good reproducibility of the instrument throughout the analysis period (Figure 5A). As expected, we observed a clear separation between the MIR and control groups, with no extreme outliers to exclude (Figure 5A), suggesting that MIR injury induces a dysregulated lipidomic profiling. Importantly, the PCA scoring plots also showed a clear aggregation of XJEK treatment with control samples (Figure 5A), indicating that the serum lipidomic dysregulation induced by MIR injury could be restored by XJEK. Such distributions and changing trends in aggregation and separation among MIR, and XJEK groups become more apparent with OPLS-DA analysis (Figure 5B). To further verify the above observations, we performed a permutation procedure test using the OPLS-DA model with the same number of component. In total, 200 rounds of random permutations of the y variable were performed, and the results showed that while the R2 values (R2 represents the validity of the model and indicates the goodness of fit) were largely steady, the Q2 values which represents the accuracy of the model prediction were substantially decreased with increasing cycles of interaction validation (Figure 5C). We found that both goodness-of-fit parameters (R2 and Q2) calculated for the ranked data were lower than the corresponding original points on the right-hand side (1 on *X*-axis), and the Q2 regression line had an intercept of 0.00897, indicating little overfitting in the original prediction model. Therefore, these analyses show that the separation model is statistically valid, and that the high value of predictability is not caused by overfitting.

The variable importance in projection (VIP) plot with jack-knifed confidence intervals based on the OPLS-DA analysis and the univariate test showed that nearly 200 lipid metabolites were significantly changed in XJEK group compared to MIR group (Figure 5D). Importantly, we observed similar changes of the serum lipidomic profiling in negative ionization (ESI-) model (Figures 5E–H).

### 3.5 Metabolic pathway analysis

**FIGURE 6:**
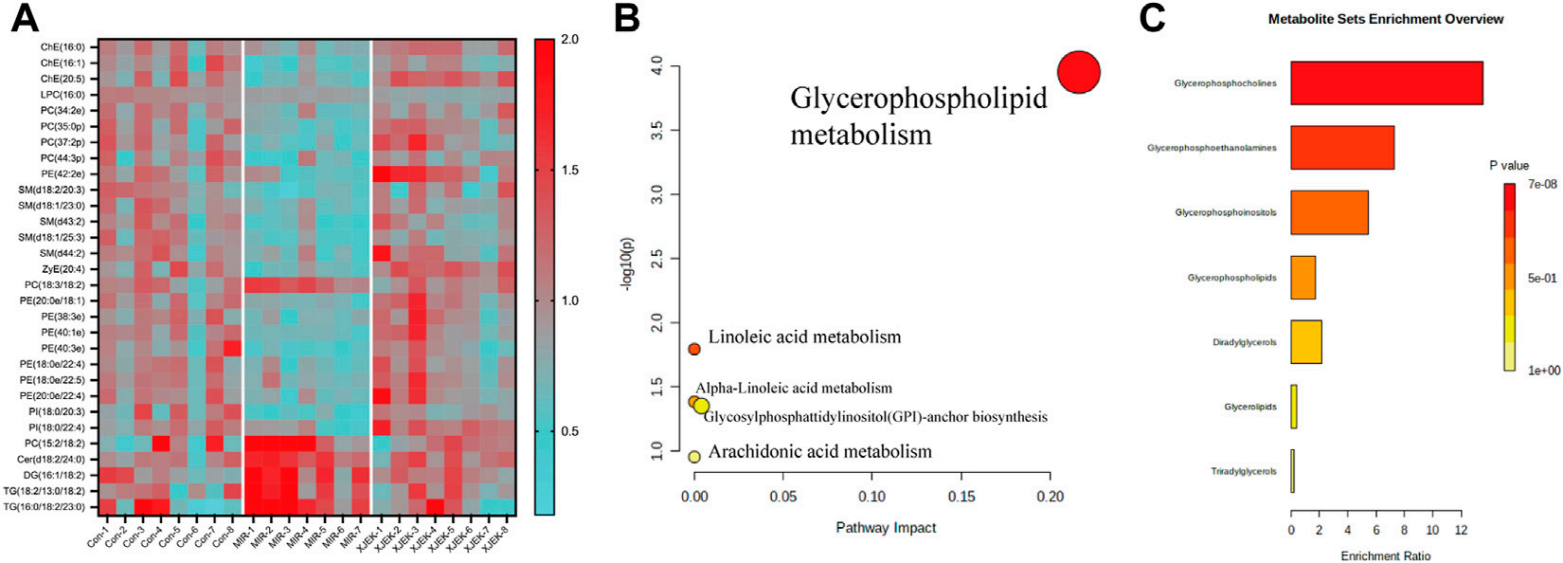
XJEK corrected dysregulated lipid metabolism. **(A)** Heatmap of hierarchical clustering analysis showing differentially expressed lipids among all the groups. The horizontal axis represents the samples; the vertical axis represents the lipid metabolites. Pathway **(B)** and enrichment **(C)** analysis based on differentially expressed lipids by MetaboAnalyst software.

To further clarify the possible metabolic pathways that were affected by XJEK, the differential lipids then were screened based on following criteria: 1) the variables with VIP value more than 1 is an important screening parameter; 2) the *p* value less than 0.05 is considered as a significant difference in the candidate lipids, both between the Control group and MIR groups, MIR groups and XJEK groups; Specifically, over 600 lipid metabolites were profiled in our samples, with 30 lipid metabolites including ceramide (CER), diacylglycerol (DG), phosphatidylcholine (PC), phosphatidylethanolamine (PE), phosphoinositide (PI), triacylglycerol (TG) and sphingomyelin (SM) were significantly altered by MIR injury (Figure 6A). We observed that all TG and DG were significantly increased, while other lipid metabolites including PC, PE and PI were dramatically decreased in MIR group compared with control group. Importantly, except LPC (16:0), 29 of the 30 MIR-altered lipid metabolites were at least partially restored by XJEK treatment (Figure 6A). Metabolism pathway analysis using the MetaboAnalyst platform in combination with KEGG databases showed that these altered lipid metabolites could be mapped into 5 metabolic pathways, with the glycerophospholipid metabolism pathway showing the largest impact and highest significance (Figure 6B). Moreover, enrichment analysis revealed a significant enrichment in metabolites associated with glycerophosphocholine, glycerophosphoethanolamine, and glycerophosphoinositol metabolism (Figure 6C).

## 4 Discussion

In this study, we have provided clear evidences to show that XJEK protects heart from MIR injury, as evidenced by improving cardiac function and reducing cardiac pathological remodeling in MIR mice with XLEK treatment. Moreover, by using high resolution Orbitrap mass spectrum we detected over 600 lipid metabolites including glycerides, phosphoglycerides and sphingolipids in mouse serum, and found apparent lipid dysregulation induced by MIR. Importantly, the altered profiling of lipid metabolites were shifted back toward to the normal levels by XJEK treatment. These data clearly suggested that XJEK exerts its cardioprotective effect upon MIR injury by correcting lipid dysregulation.

Dysregulation of lipid metabolism had been reported in ischemic and acute heart diseases, and was regarded as one of the leading causes of the development of cardiovascular disorders. Indeed, we found apparent changes with the content of the phosphoglycerides, glycerides and sphingolipids in MIR mice. Specifically, increased ceramides and sphingolipids were observed after MIR injury, which is consistent with the previous findings that elevated serum ceramides were proved to play causative roles in heart disease and could be treated as the biomarker of cardiovascular disease (Choi et al., 2021). The increasement of ceramides after ischemic injury could lead to cardiomyocyte apoptosis by inducing reactive oxygen species formation and promoting cytochrome c release (Obeid et al., 1993). Moreover, accumulation of ceramides in the mitochondrial could disrupt electron transport chain activity, thereby impairing mitochondrial energetics and inducing cell apoptosis (Hammerschmidt et al., 2019). In this study, we found that XJEK treatment could downregulate ceramide content in the context of MIR injury, explaining the beneficial effect of XJEK on MIR injury.

Moreover, both TG and DG, the representative glycerides, were found to be altered by MIR in our study. Hypertriglyceridaemia is often associated with atherosclerosis and cardiovascular diseases (Raposeiras-Roubin et al., 2021). It has been suggested that there is a close relationship between high triglyceride and MIR injury, as evidenced by accumulated TG in the circulation or heart tissues with ischemia cardiac injury (Balling et al., 2020). A similar finding was observed in our MIR mice. It has been well-known that high concentration of TG is the potential detrimental factor for cardiomyocyte injury. Even the TG itself may not lead to direct cytotoxic, the fatty acids come from TG hydrolyzation process can lead to lipotoxicity. The free fatty acids that entered into lean tissues could activate multiple pathways including oxidative stress or endoplasmic reticulum stress, then inducing cell death (Han, 2016). Importantly, we found that XJEK treatment could bring the increased TG and DG back to a normal level, which could also attribute to its beneficial effect on MIR-induced cardiac injury.

Interestingly, most of the lipids involved in the *de novo* synthesis of phospholipids were found to be decreased after MIR surgery. Phospholipids including PC, PE, PS, and PI are a general term for lipids containing phosphate group. They are the most substantial lipids which constitute the basic structures of cellular membranes including nuclear and plasma membranes. Most phospholipids are proved to be critical for the stability of cellular plasma membrane and mitochondrial membrane (Han, 2016). As such, alterations of phospholipids could severely impact cellular structures, stability and functions. For instance, decrease of mitochondrial PE content in mice has been shown to be harmful on cell survival, oxygen consumption, and ATP production (van der Veen et al., 2017). Moreover, PC has also been considered to be an independent risk factor for atherosclerosis (Kuschner et al., 2021). In the current study, we found apparent decline of serum phospholipids in MIR mice, which was reversed by XJEK treatment. Decreased phospholipids could lead to the shortage of phospholipids for cardiomyocytes in maintaining their cell membrane integrity and functions, thereby causing cardiac injury. Therefore, reverting the MIR-induced dysregulated glycerides and phosphoglycerides by XJEK may partially explain the therapeutic effects of XJEK on MIR injury.

Lipid dysregulation has a well-established role in modulation of inflammatory reactions after MIR injury. Phospholipase A2, inflammasome and NF-κB pathways have been indicated in MIR-induced inflammation. In consistent with this finding, we have observed that both serum IL-1β and TNF-α levels were significantly induced by MIR, which were completely blunted by XJEK treatment. Among numerous modified lipids we detected, TG may have the closest relationship with inflammation. TG, and their specific transporters like ApoC3, could participate in the inflammation by upregulating the pro-inflammatory genes encoding cytokines and increasing expression of leukocyte activation markers (Zewinger et al., 2020). Moreover, several inflammatory inducers such as LPS, IL-1β, or TNF-α were reported to increase TG lipolysis in liver, muscle and adipose, thus increasing serum TG (Lewis and Hegele, 2022). Furthermore, it has been recently suggested that accumulation of arachidonate 12-lipoxygenase (ALOX12)-dependent 12-HETE exacerbates MIR-induced cardiac inflammation and injury by suppressing AMPK signaling pathway (Zhang et al., 2021). Accordingly, we could speculate that XJEK protects cardiac tissues from MIR injury partially through suppressing inflammation.

## 5 Conclusion

In the current study, we have demonstrated a therapeutic potential for XJEK in MIR injury. Moreover, by utilizing non-targeted Orbitrap-MS methodology we have revealed a dysregulated serum lipidomic profile upon MIR injury. Importantly, we also provide clear evidence to support that XJEK exerts its profound anti-inflammatory and cardioprotective effect after MIR injury through suppressing inflammation and restoring the dysregulated lipid metabolism.

## Data Availability

The original contributions presented in the study are included in the article/supplementary material, further inquiries can be directed to the corresponding authors.
